# Non-Parental Family Members as Brokers of Family Social Capital: Compensatory Time Use in India

**DOI:** 10.3390/socsci9120217

**Published:** 2020-11-26

**Authors:** Melissa Alcaraz, Ashley Larsen Gibby, Nancy Luke

**Affiliations:** 1 Department of Sociology, The Ohio State University, Columbus, OH 43210, USA; 2 School of Family Life, Brigham Young University, Provo, UT 84602, USA; 3 Department of Sociology and Criminology, The Pennsylvania State University, State College, PA 16801, USA

**Keywords:** social capital, time use, adolescents, siblings, extended family, resource dilution theory, non-resident parents, India

## Abstract

Non-parental family members are understudied but important brokers of family social capital, especially in contexts without a nuclear-family norm. We used rich time diary data from a sample of 1568 South Indian adolescents to examine the relationships between any time spent with parents, parents’ residency status, and the time spent with non-parental family members. We found that adolescents with at least one non-resident parent spent significantly more time with siblings, on average, when compared to adolescents with resident parents. We further found that adolescents spent more time with siblings in educational activities, such as studying, when they had at least one non-resident parent. These findings point to the importance of considering non-parental family members in studies of family social capital, especially in low- and middle-income contexts. Our findings challenge resource dilution theories by demonstrating that siblings themselves act as resources, rather than simply competitors for parental resources.

## 1. Introduction

Social capital—a measure of individuals’ social connections and the actual or potential resources that are made available through them—is positively associated with children’s social, emotional, and educational outcomes (Coleman 1988; Dufur et al. 2008; Parcel et al. 2010; Portes 1998). Family social capital—a type of social capital that key family members provide children—is also related to child well-being (Crosnoe 2004). Previous research on family social capital has largely focused on parental investments in children; yet non-parental family members, such as siblings and grandparents, could also provide social capital to children independently or by stepping in, or compensating, when parents are unavailable (Dunifon et al. 2018; Trinitapoli et al. 2014; Geurts et al. 2015). For example, many parents have limited time to engage with their children daily. In addition, parents who do not reside with their children on a temporary or permanent basis could be at a disadvantage in creating meaningful interactions with their children. Non-parental family members could compensate for loss of parental time and thereby create family social capital for children as well.

The study of family social capital has historically focused on Western contexts, especially the United States (e.g., Calarco 2014; Cheadle 2009; Coleman 1988; Lareau 2002; Portes 1998). Family social capital could be important in low- and middle-income countries (LMICs) as well, where opportunities for upward social mobility are varied. In these contexts, non-parental family members are more likely to be important brokers of social capital, given that they are often more accessible and their interactions with children are more frequent than in the United States. For example, contrary to the nuclear-family norm, extended family members’ roles are likely more institutionalized in contexts where they often live in the same household or in close proximity to children. To date, research on family social capital has been extremely limited in LMICs in general and with respect to the role of non-parental family members in particular.

To address these gaps in the literature, we studied compensatory family social capital from non-parental family members in South India, where strong extended-family ties are common and these family members often live in close proximity (Chadda and Deb 2013; Mullati 1995). We drew from rich time diary data from a sample of adolescents (*n* = 1568) surveyed during the period 2015–2016 as part of the South India Community Health Study (SICHS). Given this understudied area of research, we employed descriptive methods as a first step to establish patterns of compensatory time use with children. We examined relationships between parental availability to children and the time investments of non-parental family members, including siblings and grandparents. In addition to the amount of time these individuals spend with children, we also explored the types of activities that siblings engage in to shed new light on the ways in which compensatory family social capital could be produced.

## 2. Background

### 2.1. Family Social Capital

The study of social capital is commonplace within studies of child well-being, schools, and families (for reviews, see Mouw 2006; Portes 1998). Social capital refers to the connections, opportunities, and knowledge made available through social ties (Coleman 1988) or, as Bourdieu stated, “the aggregate of the actual or potential resources which are linked to possession of these social networks” (Bourdieu 1986, p. 88). Social capital has important implications for the reproduction of inequality, as social capital is not evenly distributed across the population (Westin 2015). Within the sociological literature, studies focus on the positive outcomes from individuals’ personal and professional connections. Critics have also revealed the potentially negative consequences of social capital, namely the “exclusion of outsiders, excess claims on group members, restrictions on individual freedoms, and downward leveling norms” (Portes 1998, p. 15). In our study, we focus on social capital as access to the valued resources and knowledge that arise from individuals’ social networks while acknowledging the potential downsides of key relationships or the lack of access to important connections.

Family is the first social context and site of socialization that most individuals experience. Unequal access to material resources, family time, and education begins at early stages of the life course and sets individuals on stratified trajectories with long-term implications for their future well-being (Bradley and Corwyn 2002; Kalil et al. 2012). Unlike social capital, which focuses on social connections more broadly, family social capital refers specifically to familial connections that promote well-being, provide key forms of socialization, and monitor activities (Dufur et al. 2008; Parcel et al. 2010). The study of family social capital has increased in recent decades, including important work on how children spend their time and how parents implicitly and explicitly teach their children to interact with brokers of power, including teachers and healthcare professionals (Calarco 2014; Lareau 2002).

Despite this emerging interest, the study of family social capital has generally focused on interactions between parents and children, largely sidelining siblings, grandparents, and other non-parental family or household members (Crosnoe 2004; Dufur et al. 2008; Furstenberg 2005). This is partially due to a narrow research emphasis on the United States (Lareau 2002; Nauck 2001; Parcel et al. 2010), where nuclear families made up of co-residing parents and children are the norm and non-parental family members are, on average, not highly involved (Pew Research Center 2010, 2015). Nevertheless, there is a diversity of family structures in the United States across socioeconomic status and racial and ethnic groups (e.g., Barnett et al. 2016). Many minority households are multi-generational or have extended kin living in close proximity, which is often the case across LMICs as well (e.g., Zeng and Xie 2014).

### 2.2. Non-Parental Family Members and Family Social Capital

The literature on non-parental family social and economic support highlights the roles of two types of family members: grandparents and siblings. Grandparents are key care providers within multi-generational households, often assisting their adult children with raising their sons and daughters (Chen et al. 2011; Fuller-Iglesias 2015; Turney 2014). Studies have shown that older family members collectively parent children across the globe (e.g., Chadda and Deb 2013). Siblings, on the other hand, are often viewed as competitors for family resources, which negatively impacts outcomes, as resources become diluted once distributed across siblings. For example, larger sibship size is generally associated with lower educational performance and attainment among children (Downey 1995; Gibbs et al. 2016).

In contrast to resource dilution arguments, multiple studies have found that siblings provide valuable resources to each other and the entire family. For example, in many parts of sub-Saharan Africa, older siblings often pay for their younger siblings’ education fees and house younger siblings for secondary or tertiary education in urban locations (Buchmann and Hannum 2001; Lloyd and Blanc 1996; Lloyd and Gage-Brandon 1994; Trinitapoli et al. 2014). In other contexts, siblings also pass on social and interpersonal skills to each other (Downey and Condron 2004). In addition, siblings often take on parental roles and responsibilities in large families or when parents are absent due to migration, for example (e.g., Titzmann 2012).

The research on sibling and grandparent support suggests that non-parental family members could be important sources of family social capital for children. Their involvement could substitute or compensate for parental investments and interactions with children when parents are not available. Thus, we expect that children’s involvement with non-parental relatives, particularly siblings and grandparents, increases when parents are not available.

### 2.3. Time Use as a Dimension of Family Social Capital

Measures of family social capital vary widely and generally aim to gauge family connections and cohesion (for a review, see Alvarez et al. 2017). Coleman (1988) identified two dimensions of family social capital—the actual physical presence of parents and the quality and frequency of parental interactions with children. Previous scholars have measured the presence or absence of parents in the home with constructs such as parents’ marital status and usual weekly work hours (Parcel and Dufur 2009). The quality and frequency of parent–child interactions have been measured by parents’ knowledge of children’s activities, the home environment, parents’ support of and expectations for children, emotional closeness between parents and children, and beliefs about the importance of children spending time with their families (Behtoui 2017; Crosnoe 2004; Daly 1996; Parcel and Dufur 2009).

One line of research has focused on the actual time parents spend with children as well as the types of activities they engage in as detailed measures of family social capital (e.g., Bianchi and Robinson 1997). Time together could indicate opportunities to create and maintain emotional bonds and transfer information (Arnett 1995; Daly 1996). Time spent with children in specific activities, such as helping with homework, could also signal higher quality interactions. For example, several studies have found that children benefit emotionally and cognitively from time with parents, particularly when that time is spent in engaging or intentional activities, such as reading or educational play (e.g., Cano et al. 2019; Mitchell et al. 2009; Pleck 1997). In addition, temporary or permanent parental absence could significantly constrain time with children, and prior research has found that child outcomes, like academic performance or connections with other family members, are negatively affected by parental absences due to incarceration or divorce, for example (Turney 2014; Sun and Li 2009).

With respect to non-parental family members, research has shown that grandparents and older siblings spend time with children in numerous activities, including play, eating meals, media use, assisting children with schoolwork, organizing children’s leisure time by scheduling extracurricular activities, and having extended conversations (e.g., Dunifon et al. 2018; Lareau 2002). These studies do not relate such non-parental time to parental availability, however, to gauge possible compensatory behavior.

### 2.4. The Present Study

We bridge existing gaps in the literature by examining whether non-parental family members compensate for parental absence from adolescent children by increasing their time spent with these adolescents. Our study is set in South India, where social capital, including family social capital, is increasingly important as educational and occupational competition grows, and social capital is needed for success in the labor and marriage markets (Chatterjee et al. 2018). Furthermore, social and economic contexts in South India encourage close, extended family ties. For example, endogamous marriage, marrying within someone’s social or kin group or within the same village, is relatively common (Dyson and Moore 1983). Consequently, brides and grooms often live in the same village (Kapadia 1998), and extended family members remain geographically close after marriage. This means that families in South India have ample opportunity for non-parental compensatory behaviors, making it a useful study site.

We use detailed time diary data to measure adolescents’ time spent with specific family members and in various types of activities. The measurement of time spent with children enables us capture both dimensions of family social capital as defined by Coleman (1988): the actual physical presence of key household members and the frequency of their interactions. By spending time with children and engaging in supportive types of activities, we argue that non-parental family members could compensate for decreased parental availability.

The time use data allow us to answer two main research questions: (1) do non-parental family members, particularly grandparents and siblings, compensate for decreased parental availability for adolescent children? (2) If so, which activities do non-parental family members engage in with adolescents? Given that non-parental social capital in LMICs is an understudied area, we employed descriptive methods to investigate the overall patterns of the quantity and quality of time non-parental family members spend with adolescents.

## 3. Data and Method

### 3.1. Data and Sample

We utilized survey data from the South India Community Health Study (SICHS), a data collection project in 400 villages in rural Vellore District, Tamil Nadu, South India. SICHS included two data collection efforts: a census of 300,000 households and a random sample of ever-married men aged 25–60 as primary respondents. The random sample of primary respondents also includes a small number of divorced or widowed women whose husbands would have been between the ages of 25 and 60, based on the average age-gap between husbands and wives. The SICHS sample is representative of each caste in the study area, aside from castes with less than 100 households in the census. Each randomly sampled primary respondent was interviewed, along with their spouse, if any, and their adolescent child(ren), aged 12–17, if any. The household response rate was over 85%. The SICHS sample is reflective of socioeconomic and demographic characteristics of rural Tamil Nadu and rural South India (Borker et al. 2019).

These data are particularly well suited to answer questions about family time use as they include adolescent self-reported 24-hour time diary data. Adolescents were asked to list, hour by hour, each activity they participated in during the preceding day. They started their reporting at midnight the day before and went through 24 hours. Activities were allowed to overlap (i.e., respondents could multi-task), and they reported with whom they were engaged for each activity, if anyone. Time diary data are often considered the best data collection method for time use because they have been found to provide precise estimates (Gershuny and Robinson 1988; Sayer et al. 2004). Time diaries pose fewer memory demands and are less vulnerable to self-concept biases than survey questions asking respondents how much time they spend in certain tasks, or with certain people, on an average day or week.

Our sample included adolescents aged 12–17 who completed the questionnaire (*n* = 1568). As shown in Table 1, in our sample, 91% of adolescents resided in a two-parent family, 58% had at least one parent who completed primary education (completion of Standard 8 (S8)), and the average household income was approximately 12,721 Indian rupees (INR) per month (roughly USD 195). About 1 in 4 adolescents lived with a grandparent, whereas almost all lived with a sibling (99%) and very few lived with an aunt/uncle (3.45%) or a cousin (0.83%). Most adolescents in the sample (69%) were the oldest children with an average of 2.5 siblings. Half of the sample was female (49%), 14 years old on average, and most adolescents (80%) reported time diary information on a weekday.

### 3.2. Measures

Using the time diary data, we observed the amount of time adolescents reported spending with different family members including mothers, fathers, grandparents, siblings, aunts/uncles, and cousins. Both resident and non-resident family members were included. We summed the number of minutes adolescents reported spending with their respective family members across all activities.

For parents, we dichotomized time investments to measure whether adolescents reported spending any time with their mothers (1 = any time with mother, 0 = no time with mother), their fathers (1 = any time with father, 0 = no time with father), or either parent (1 = any time with either parent, 0 = no time with either parent). Dichotomizing these measures allowed us to observe the presence or lack of parental availability, and hence, social capital formation.

We estimated whether at least one parent was currently a non-resident of the household as an additional measure of parental availability. Primary respondents were asked if each household member was “temporarily absent” on a household roster. We measured parents’ residency status using this item (1 = at least one parent was temporarily absent, 0 = no parent was temporarily absent).

In terms of non-parental family members, we measured the number of minutes adolescents reported spending with their siblings overall, regardless of activity, and across five types of activities. First, we measured time spent with a sibling in recreational activities, which included time spent playing video games; being on the computer/internet or social media; listening to music; reading books, magazines or newspapers; attending the cinema or theater; participating in outdoor sports or other forms of exercise or indoor sports or play; caring for or playing with pets; talking on the telephone or mobile phone; and relaxing. Our second activity included time watching TV, including videos and movies, and the third activity was time spent socializing or chatting. Fourth, we measured time spent in educational activities, including time in courses/school/college, study time/homework, tuitions (tutoring sessions), and extracurricular school activities (not including sports). Finally, we included a measure of time spent travelling by any means of transportation (e.g., car, bus, train, bicycle).

### 3.3. Analytic Strategy

Our study is a descriptive analysis of non-parental family time investments by the availability of parental social capital. We observed the amount of time adolescents spent with grandparents, siblings, aunts/uncles, and cousins by whether adolescents spent any time with their mothers, fathers, or either parent, as well as by whether adolescents had a non-resident parent or not. Based on these initial analyses, we then focused on sibling time and estimated the amount of time adolescents who had at least one sibling reported spending with their siblings across various activities by parents’ residency status.

## 4. Results

Our results show that adolescents spent different amounts of time with family members by parental availability (Table 2). For example, adolescents who spent any amount of time with their mothers spent significantly more time with their siblings, on average, than adolescents who spent no time with their mothers (297 daily minutes versus 216 daily minutes), and this difference was statistically significant (*p* < 0.01). We see similar patterns with sibling time across the time spent with fathers and with either parent. In short, when adolescents spent time with any parent, they spent more time with their siblings. We see a reversal in this relationship when observing differences in parental residency status. Here, adolescents spent significantly more time with their siblings, on average, when they had a non-resident parent (341 daily minutes) than adolescents who had resident parents (280 minutes), and this difference was statistically significant (*p* < 0.05).

With respect to other family members, adolescents who spent no time with their mothers spent significantly more time with their aunts and uncles (12 daily minutes), on average, than adolescents who spent some time with their mothers (5 daily minutes). In contrast, adolescents who spent no time with their fathers spent significantly less time with their aunts and uncles, on average, than adolescents who spent some time with their fathers (3 daily minutes versus 8 daily minutes). Furthermore, we did not find significant differences in time with grandparents or cousins by parental availability.

Our results revealed a pattern of compensatory time with siblings when at least one parent was a non-resident. We carried out a further analysis of the types of activities siblings engaged in by parental residency. We limited our sample to adolescents with siblings (*n* = 1558), excluding 10 adolescents who had no siblings (Table 3). We found no statistically significant differences in adolescents’ time with siblings in recreation, TV viewing, socializing/chatting, or travel by parental residency status. There was a significant difference, however, in educational time. On average, adolescents who had at least one non-resident parent spent more time with a sibling (55 daily minutes) than those who had resident parents (38 daily minutes) in school, extracurricular activities, or study time.

## 5. Discussion

Previous research has conceptualized family social capital almost exclusively as parental social capital (Coleman 1988; Crosnoe 2004; Furstenberg 2005; Parcel et al. 2010). Although scholars have found that parents are valuable brokers of social capital, we argued that the focus on parents obscures the meaningful roles of non-parental family members. This could be especially important in contexts that do not hold the nuclear family norm. We examined an innovative measure of family social capital—time use—across different family members of adolescents in South India and how these time investments varied by parental availability. Measures of time in specific activities can shed light on when and how family social capital is formulated for children. We contributed to the current literature by examining family social capital in a non-western, LMIC context with strong extended family ties, focusing on non-parental brokers of family social capital, and examining compensatory family social capital. We further utilized rich, unique data from adolescent self-reported time diaries.

The time diary data allowed us to explore the quantity and quality of time adolescents spend with non-parental family members, and our study produced four main findings. First, our results suggest that non-parental family members, particularly siblings, compensate for the loss of family social capital due to parents’ absence in the home. South Indian adolescents with at least one non-resident parent spent more time with siblings, on average, than those with resident parents. This is consistent with past studies focused on high-migration contexts, for example, which have found that siblings provide both material resources and emotional support for other members of the household when parents are not present (East 2010; Titzmann 2012). While we have no information on the reasons for temporary parental non-residence in our data, labor migration or short-term family obligations, such as caring for an ill relative, are likely options (Morten 2019). Second, we found that adolescents with at least one non-resident parent spent significantly more time with siblings in educational activities, on average, compared to adolescents with resident parents. This suggests that siblings’ time spent together in these activities is an important source of family social capital that could be linked to subsequent educational outcomes (e.g., Dufur et al. 2013; Wu et al. 2014).

These findings also challenge resource dilution theory (Downey 2001; Gibbs et al. 2016; Steelman et al. 2002), which views siblings as drains on parental resources rather than useful resources themselves. In South India, siblings appear to create family social capital, not dilute it. The role and contribution of siblings is likely context dependent, however. In settings where siblings serve as substitute caregivers and often provide a high level of social and material support, such as India, siblings’ contributions could be considerable. In contrast, in high-income settings with strong boundaries between parental and child roles, siblings may be less prepared to become brokers of family social capital. Taken together, these findings support our contention that siblings are influential and understudied sources of family social capital that should be investigated in future research.

Third, we measured parents’ availability to adolescent children in two ways, as any time with adolescents and as co-residency, which revealed contrasting findings. On the one hand, adolescents who spent no time with their mothers and fathers spent significantly less time with siblings, on average, than adolescents who spent any amount of time with mothers or fathers on the time diary day. Rather than substituting for lost parental time, siblings spent more time with adolescents when parents were also present. In this case, it could be that parents encourage more family time together, or that families who work in agricultural settings tend to work together more often, for example (Clark et al. 2017, 2019; Madhavan and Gross 2013). On the other hand, as noted above, we found that siblings spent more time with adolescents when at least one parent was a non-resident. These findings suggest that siblings compensate time when there is a significant deficit of parental availability, such as through temporary absence from the home.

Fourth, grandparents, aunts and uncles, or cousins did not consistently differ in the time spent with adolescents by parental availability, and therefore did not display compensatory behavior. This is somewhat surprising, considering the greater involvement of extended family members—particularly grandparents—in Asia compared to the United States, for example (Settles et al. 2009). One potential explanation is that non-parental family members do not compensate in terms of time but through other forms of family social capital, such as connecting young adults with potential employers, or through economic capital, by providing financial resources for education or tutoring. Another possible explanation is that extended family members do not compensate for parental absence in terms of more time with adolescents but in the types of activities they engage in. For example, grandparents could switch from spending time with adolescents in recreation to spending that time in educational activities when parents are absent.

Our study had several limitations. First, some researchers may hold the view that descriptive analyses are not comprehensive examinations. We argue that our descriptive results illuminate broad patterns and relationships, which are essential in an under-researched area. Indeed, our results are quite informative and may drive multiple avenues of future research. For example, follow-up investigations could focus on specific family members, such as siblings, and determinants of compensatory time with adolescents, such as the duration of parental absence, in more complex models. Future research should also consider how compensatory family social capital measured by time diaries is related to educational, emotional, and social outcomes; whether certain family members are more beneficial for children for specific outcomes; and the context-specific nuances to these relationships.

Second, our analyses did not consider important variations by gender, race, class, caste, or their intersections. In the United States, for example, people of color are more likely to reside in multi-generational households than whites (Cohn and Passel 2018), as are those of lower socio-economic statuses and immigrant families (Pew Research Center 2010). Therefore, whether and how non-parental family members create and compensate family social capital could differ substantially across these dimensions and have important implications for narrowing or increasing inequalities in outcomes among adolescents.

Third, our study was limited in some lines of inquiry due to data constraints. For example, we were not able to assess the mood or tone of time spent with family members, which could be negative or contentious rather than positive and conducive to building emotional connections and family social capital. Future research investigations and new data sets could help answer these important questions.

In conclusion, our study represents a key first step in incorporating non-parental family members into the family social capital discourse. Our analysis of time diary data from adolescents in South India produced interesting descriptive patterns of the quantity and quality of time non-parental family members spend with adolescents and how this time varies by parental availability. Among the most notable findings was the importance of siblings as brokers of family social capital. Siblings can serve as resources for adolescents and appear to compensate for shortfalls in parental social capital.

## Figures and Tables

**Table 1.** Descriptive Statistics (Means and Percentages) for Adolescents, Tamil Nadu, India (*n* = 1568 Adolescents).

Household Characteristics	Means or Percentages

Non-resident mother	0.19
Non-resident father	9.13
Family structure	
Single mother	7.02
Single father	1.98
Two-parent family	91.00
Any parent has over S8 education ^a^	58.08
Household monthly income in INR (mean) ^b^	12,720.72

Adolescent Characteristics	

Resident grandparent	24.27
Resident sibling	99.36
Resident aunt or uncle	3.45
Resident cousin	0.83
Birth order (mean)	1.34
Oldest child	69.35
Number of siblings (mean)	2.52
Female	48.79
Age (mean)	14.36
Interviewed on a weekday	79.73

Data: South India Community Health Study.

a. S8 = completed Standard 8 (primary education).

b. INR = Indian rupee; 1 USD = INR 65 in 2015–2016.

**Table 2.** Minutes Spent with Family Members across Different Parental Availability Measures (*n* = 1568 Adolescents).

	Mother		Father		Either Parent		Parental Co-Residence	
Minutes with	No Time	Any Time	No Time	Any Time	No Time	Any Time	Non-Resident Parent	Resident Parents
Grandparents	23.73	22.06	26.31	16.17	24.16	12.33	23.76	23.50
Siblings	215.86	296.57 **	246.56	300.03 *	131.14	294.36 ***	341.34	279.87 *
Aunts/uncles	11.95	5.53 *	2.86	7.80 *	4.09	6.57	4.93	6.59
Cousins	11.00	11.53	12.33	9.18	1.48	12.05	10.93	11.52

Data: South India Community Health Study.

* *p* < 0.05

** *p* < 0.01

*** *p* < 0.001; determined by *t*-tests.

**Table 3.** Minutes Spent with Siblings across Different Activities by Parental Residency (*n* = 1558 Adolescents).

Minutes in	Non-Resident Parent	Resident Parents	Statistically Significantly Different
Recreation	30.78	24.79	
TV viewing	172.39	141.29	
Socializing/chatting	25.08	24.69	
Educational activities	55.22	37.81	*
Travel	15.74	11.60	

Data: South India Community Health Study.

* *p* < 0.05; determined by *t*-tests.
